# Targeting the Gut Microbiota to Treat Cachexia

**DOI:** 10.3389/fcimb.2019.00305

**Published:** 2019-09-12

**Authors:** Laurence Genton, Julie Mareschal, Yannick Charretier, Vladimir Lazarevic, Laure B. Bindels, Jacques Schrenzel

**Affiliations:** ^1^Clinical Nutrition, Geneva University Hospitals and University of Geneva, Geneva, Switzerland; ^2^Genomic Research Laboratory, Service of Infectious Diseases, Geneva University Hospitals and University of Geneva, Geneva, Switzerland; ^3^Metabolism and Nutrition Research Group, Louvain Drug Research Institute, Université Catholique de Louvain, Brussels, Belgium

**Keywords:** gut, gut microbiota, metabolism, cachexia, metagenomic

## Abstract

Cachexia occurs in many chronic diseases and is associated with increased morbidity and mortality. It is treated by nutritional support but often with limited effectiveness, leading to the search of other therapeutic strategies. The modulation of gut microbiota, whether through pro-, pre-, syn- or antibiotics or fecal transplantation, is attracting ever-growing interest in the field of obesity, but could also be an interesting and innovative alternative for treating cachexia. This article reviews the evidence linking the features of malnutrition, as defined by the Global Leadership Initiative on Malnutrition [low body mass index (BMI), unintentional body weight loss, low muscle mass, low appetite, and systemic inflammation] and the gut microbiota in human adults with cachexia-associated diseases, and shows the limitations of the present research in that field with suggestions for future directions.

## Introduction

Cachexia is “a multifactorial syndrome characterized by a progressive loss of body weight and skeletal muscle mass” (Evans et al., 2008). Although this term is mostly used in the context of cancer, cachexia can also be found in other chronic diseases as for instance chronic obstructive pulmonary disease, chronic heart, kidney, or liver failure, AIDS and rheumatoid arthritis (Muscaritoli et al., 2010). Its prevalence is estimated between 10 and 50% depending on the underlying disease (von Haehling et al., 2016). Clinical consequences of cachexia are impaired physical function, fatigue, low quality of life, longer length of hospital stay, and increased mortality (Sorensen et al., 2008), and in case of cancer, increased risk of chemotherapy toxicity with subsequent dose reductions and treatment delays (Barret et al., 2011).

Several expert consensuses and observational studies have provided clinical definitions for cachexia, mostly in the context of cancer (Table 1). In 2015, a consensus of the European Society of Clinical Nutrition and Metabolism defined cachexia as disease-related malnutrition which differentiates it from starvation, sarcopenia and frailty (Cederholm et al., 2015). More recently, the Global Leadership Initiative on Malnutrition (GLIM), which involves experts of several nutritional societies from all over the world, recommended to diagnose malnutrition with at least one phenotypic criterion [low body mass index (BMI), unintentional weight loss, or decreased muscle mass] and one etiologic criterion (reduced food intake or assimilation, disease burden or inflammatory state) (Cederholm et al., 2018; Jensen et al., 2019). Although some features of cachexia may differ from other types of malnutrition (for instance the inherent inclusion of disease burden in cachexia), the authors recommend using these clinical diagnostic criteria whatever the type of malnutrition, as the priority is to determine whether a patient needs nutritional support.

**Table 1:** Definitions of cachexia.

**References**	**Criteria for cachexia**	**Criteria basis**
Fearon et al., 2006	Cachexia in pancreas cancer: Unintentional weight loss ≥10% in the last 6 months and Decreased energy intake ≤1,500 kcal and Systemic inflammation (CRP ≥ 10 mg/l)	Single center study comparing weight loss vs. weight loss + decreased energy intake + systemic inflammation on body composition, functional status and survival
Evans et al., 2008	Cachexia: Unintentional weight loss ≥ 5% in the last 12 months and 3 of the following critera: Reduced muscle strength Reduced fat-free mass index Fatigue Biological abnormalities: increased inflammatory markers, anemia, hypoalbuminemia	Expert consensus
Bozetti and Mariani, 2009	Cachexia in cancer: Unintentional weight loss ≥10% in the last 6 months and Anorexia or Fatigue or Early satiation	Single center study evaluating the trend of clinical nutritional and oncologic variables between 4 classes of severity of cachexia, based on combinations of unintentional weight loss + anorexia, fatigue or early satiation
Muscaritoli et al., 2010	Pre-cachexia: Unintentional weight loss ≤ 5% in the last 6 months and Underlying chronic disease and Chronic or systemic inflammatory response and Anorexia or anorexia-related symptoms	Expert consensus
Fearon et al., 2011	Pre-cachexia in cancer: Unintentional weight loss ≤ 5% in the last 6 months and Anorexia Cachexia in cancer: Weight loss > 5% in the last 6 months or Body mass index <20 and weight loss >2% or Sarcopenia and weight loss >2% Often reduced food intake/systemic inflammation Refractory cachexia in cancer: No answer to treatment and Low performance score and <3 months survival	Expert consensus
Argilés et al., 2011	Cachexia score (CASCO) in cancer: Unintentional weight loss and lean body mass loss (40%) Anorexia (15%) Inflammatory, immunological and metabolic disturbances (20%) Physical performance (15%) Quality of life (10%)	Expert consensus, score not yet validated
Martin et al., 2015	Five stages of cancer cachexia according to: Unintentional weight loss Body mass index	Multicenter cohort study evaluating the impact of body mass index and weight loss on survival

Presently, cachexia is treated by nutritional support, but with limited effectiveness (Aquilani et al., 2008; Baldwin et al., 2012; Ferreira et al., 2012; Konishi et al., 2016). This implies the need of a multimodal approach including not only nutritional support or orexigenic agents, but also anti-catabolic and anti-inflammatory treatments. A promising therapeutic target for cachexia may lie in the gut microbiota that interacts with the other components of the gut barrier, namely the gut epithelium, the gut-associated lymphoid tissue (GALT) and the enteric nervous system.

This article reviews the evidence linking the features of malnutrition as defined by the GLIM and the gut microbiota in human adults with cachexia-associated diseases, and shows the limitations of the present research in that field with suggestions for future directions. Although microbiota in humans is generally measured in the feces, we will refer to it as gut microbiota.

## Low BMI and Gut Microbiota

The link between overweight/obesity and gut microbiota is attracting ever-growing interest, but the association between a BMI below the normal range (<18.5 kg/m^2^) and gut microbiota in cachexia-associated diseases has not been formally evaluated.

A few studies investigated the gut microbiota by 16S rRNA gene profiling in patients with anorexia nervosa, a condition of low BMI due to starvation, i.e., negative energy balance. Their microbial alpha-diversity was low (Kleiman et al., 2015) or normal (Mack et al., 2016; Borgo et al., 2017). Compared to lean healthy controls, alterations at the phyla level are controversial, especially regarding Bacteroidetes and Firmicutes (Armougom et al., 2009; Mack et al., 2016; Borgo et al., 2017). At the family level, patients with anorexia nervosa display a lower abundance of *Enterobacteriaceae* (Borgo et al., 2017) and *Lactobacillus* (Mack et al., 2016). Studies reported a reduction in the genera *Ruminococcus, Clostridium, Roseburia* (Borgo et al., 2017), *Anaerostipes, Faecalibacterium*, and in unspecified genera of the order *Coriobacteriales* (Kleiman et al., 2015), and of the species *Clostridium coccoides, Clostridium leptum*, and *Bacteroides fragilis*. Some of these bacteria are producing short-chain fatty acids, which are key signaling molecules for the regulation of appetite, lipid and glucose metabolism as well as immune functions (Morrison and Preston, 2016). A lower fecal concentration of propionate, acetate (Morita et al., 2015) and butyrate (Borgo et al., 2017) was described. Finally, several studies found higher levels of the archaeon *Methanobrevibacter smithii* in these patients (Armougom et al., 2009; Mack et al., 2016; Borgo et al., 2017).

Two trials followed the patients with anorexia nervosa after a refeeding protocol leading to an increase of BMI by 2.3 ± 1.2/kg/m^2^ (Mack et al., 2016), and 1.2 kg/m^2^ (Kleiman et al., 2015). Gut microbiota composition of these patients did not normalize with weight gain, although their microbial diversity and richness was increased.

These studies show the large inter-individual variability of gut microbiota composition in patients with anorexia nervosa and the inconsistencies between some studies. Such inconsistencies could be attributed either to the low number of patients included in these studies or to regional variation in gut microbiota being larger than the anorexia microbial signature itself (He et al., 2018). If the gut microbiota is to play a role in body weight regulation, these results suggests that either taxonomic discrimination needs to be performed more in depth or that the bacterial function and metabolites are more important than the microbiota composition for BMI regulation. For instance, stool culture could identify 11 new bacterial species in a young anorectic patient with a BMI of 10.4 kg/m^2^ compared to 16S rRNA, which demonstrates the room for improvement in the technology for taxonomic discrimination (Pfleiderer et al., 2013). Whether the condition of anorexia nervosa can be compared to cachexia is unclear as it does not take into account other parameters of cachexia, as for instance systemic inflammation.

## Body Weight Loss and Gut Microbiota

Body weight loss is a dynamic condition requiring a longitudinal follow-up. To our knowledge, no study has concentrated on the link between unintentional weight loss and gut microbiota.

A recent systematic review including 10 studies focused on surgery-induced voluntary weight loss in humans (Guo et al., 2018). Bariatric surgery had a controversial impact on the richness and diversity of gut microbiota. It led to a decrease in the abundance of Firmicutes, *Clostridiales, Clostridiaceae, Blautia*, and *Dorea* but to an increase in Bacteroidetes, Fusobacteria, Proteobacteria, and Verrucomicrobia. Shotgun metagenomic sequencing highlighted the up-regulation of nitrogen and fatty acid metabolism, and, after Roux-en-Y gastric bypass, the stimulation of the phosphotransferase system, involved in bacterial sugar uptake, and of the sulfur relay system and the purine metabolism. When considering weight loss induced by hypocaloric diets, another systemic review found no consistent effects on microbial alpha-diversity, abundance or phylum composition (Seganfredo et al., 2017). However, this intervention induced a decrease in *Bifidobacterium* spp, *Clostridium* cluster XIVa species, *Roseburia* spp, and *Eubacterium rectale*. Both reviews showed a correlation between changes in specific bacterial taxa and metabolic issues, like improvements in lipid profile, glucose homeostasis, and inflammatory markers. However, unintentional weight loss can likely not be compared to these situations as the pathogenesis is different.

Some papers report an unintentional weight gain after fecal microbiota transplantation (FMT) in patients with chronic diseases. A case-report showed a weight gain of 17 kg in 6 months after FMT for recurrent *Clostridium difficile* colitis, in a heart-transplant patient weighing 37.9 kg at baseline (Ehlermann et al., 2014). Another case-report demonstrated a weight gain of 18 kg within 3 years after FMT for recurrent *C. difficile* colitis (Alang and Kelly, 2015). Finally, in 22 patients with Crohn's disease, an FMT led to a mean weight gain of 3 kg in 6 months (Cui et al., 2015). These findings suggest that components of the FMT, as for instance gut microbiota, could also be involved in unintentional weight loss.

Similarly, some antibiotics seem to be involved in weight gain (Angelakis et al., 2014). It was speculated that the unintentional BMI increase (2.3 ± 0.9kg/m^2^ in 1 year) induced by vancomycin/gentamicin therapy in infective endocarditis patients could be due to the gut colonization of *Lactobacillus* spp. These microorganisms, used as growth promoter in animals, are intrinsically resistant to vancomycin and were over-represented in the feces of obese patients (Thuny et al., 2010). Claritromycin was also associated with weight gain (4 kg in 3 months) in patients with non-small cell lung cancer, leading the authors to suggest that this antibiotic may limit the progression of cachexia (Sakamoto et al., 2001). In any case, whether antibiotics should be used against weight loss is very questionable in view of the thread of antibiotic resistance.

## Low Muscle Mass and Gut Microbiota

The association between low muscle mass and gut microbiota has been suggested in a few studies comparing older vs. younger people, and sedentary vs. physically active people.

Older people are known to be at risk for malnutrition, especially of sarcopenia which is defined as a low muscle mass and function. In 23 nursing home residents ≥65 years, malnutrition was associated with a lower abundance of butyrate-producing organisms (*Roseburia intestinalis* and *Subdoligranulum*) and higher loads of dysbiotic bacteria such as *Enterococcus feacalis* and *Citrobacter freundii*, as compared to a good nutritional state (Haran et al., 2018). Shotgun metagenomic sequencing highlighted a lower abundance of genes involved in vitamin B production and in the metabolism of essential amino acids, purine and pyrimidine, in older vs. younger residents, and an increased biosynthesis of the bacterial wall components peptidoglycan and lipopolysaccharides in those who were malnourished. Buigues et al. randomized 60 apparently healthy volunteers ≥65 years to a daily oral prebiotic composed of inulin and fructooligosaccharides or a maltodextrin placebo for 13 weeks. The group that received the prebiotics improved their self-reported chronic fatigue and measured handgrip strength (Buigues et al., 2016). No analysis of gut microbiota was performed. None of these studies determined muscle mass and thus the link remains speculative in older people.

Gut microbiota differences were also found between athletes and sedentary subjects. Clarke et al. compared the gut microbiota of 40 male rugby players (BMI 28.8 ± 3.8 kg/m^2^), with that of age-matched healthy controls having either a normal (22.7 ± 1.8 kg/m^2^) or high BMI (31.2 ± 3.0 kg/m^2^) (Clarke et al., 2014). Compared to the high BMI controls, the rugby players had a higher lean body mass and an increased proportion of the mucin-degrading species *Akkermansia muciniphila*. The relative protein intake of the rugby players was higher and positively correlated with microbiota diversity. The authors extended their analysis by applying metagenomic shotgun sequencing (Barton et al., 2018). They found an increase in the pathways related to amino acids and antibiotic biosynthesis and carbohydrate degradation in athletes and reported higher levels of fecal short-chain fatty acids. Interestingly, Allen et al. randomized 32 lean and obese sedentary persons to a 6-week endurance training, thrice weekly, followed by a 6-week sedentary period (Allen et al., 2018). Exercise significantly increased lean body mass, cardio-respiratory fitness and decreased fat mass but body composition of all participants returned to baseline values after the 6 weeks of sedentary lifestyle. Butyrate-producing taxa increased with exercise in lean participants and correlated with increases in lean mass and reduction of fat mass. Thus, physical exercise modulates gut microbiota although most of these studies are observational and cross-sectional. Knowing that resistance exercise is a recognized treatment to counteract loss of muscle mass and function (Deutz et al., 2014), these studies suggest a potential link between gut microbiota and muscle.

## Low Appetite and Gut Microbiota

Appetite is regulated at the level of the arcuate nucleus of the hypothalamus, more precisely in the anorexigenic pro-opiomelanocortin-expressing neurons and the orexigenic agouti-related peptide and neuropeptide-expressing neurons. The hypothalamus receives inputs from the enteric nervous system via the vagus nerve, and from appetite mediators via the peripheral circulation or the vagus nerve (van de Wouw et al., 2017). A low appetite, as in cachexia, could thus result from an altered interaction between the gut microbiota and the enteric nervous system or appetite mediators originating in the gut.

Vagotomized subjects were shown to have a decreased appetite and hunger and an increased sensation of satiety and fullness. However, no human study showed the role of gut microbiota on the vagus nerve signaling. Gut microbiota derived-short-chain fatty acids can stimulate the free-fatty acid receptor 3 (FFA3), expressed on portal nerves, and thus trigger the vagus nerve in animal studies (De Vadder et al., 2014).

Human studies start to concentrate on the link between gut microbiota and appetite mediators or appetite sensation. The anorexigenic neuropeptide PYY, the glucagon-like peptide-1 (GLP-1), both secreted by the enteroendocrine cells of the ileum and colon, and the cholecystokinin, secreted by the duodenum, could theoretically be easy targets for the gut microbiota or its metabolites. If gut microbiota modulates appetite via intestinal appetite mediators, high levels of PYY and GLP-1 and dysbiosis would be expected in cachexia, but this relationship has not yet been explored. In overweight or normal-weight subjects, several studies evaluated the impact of prebiotics, known to modulate the gut microbiota, on plasma PYY and GLP-1, although with controversial effects (Cani et al., 2009; Klosterbuer et al., 2012; Chambers et al., 2015). Reimer et al. showed an increase abundance of *Bifidobacterium* and a lower appetite in overweight adults assigned to inulin-type fructans intake for 12 weeks, as compared to whey protein or nothing (Reimer et al., 2017). Recently, a randomized open-label trial exposed patients with type 2 diabetes mellitus to a high-fiber diet or a conventional educational and dietetic program over 3 months (Zhao et al., 2018). The high-fiber diet led to a higher weight loss, higher plasma levels of post-prandial GLP1 and fasting PYY, and an increase in fecal butyric acid. These results suggest that dietary fibers may regulate the level of appetite mediators through short-chain fatty acids production.

Interestingly, among the orexigenic mediators, the ghrelin-receptor agonist anamorelin, given at 100 mg once a day for 12 weeks was shown to increase lean body mass in two studies including cachectic patients with non-small lung patients (Takayama et al., 2016; Temel et al., 2016). Whether gut microbiota composition or function inhibits ghrelin secretion from the stomach or pancreas in cachectic patients is unknown.

## Systemic Inflammation and Gut Microbiota

The GALT is the largest immune organ. The complex association of the gut microbiota with systemic inflammation in the context of obesity and high-fat diet is beyond the scope of this paper (Nagpal et al., 2016).

An association between systemic inflammation and gut dysbiosis has been shown in cachexia-associated diseases as chronic kidney disease (Kanbay et al., 2018), hepatic liver cirrhosis (Ahluwalia et al., 2016), and heart failure (Nagatomo and Tang, 2015). If gut microbiota was to be a causal factor of the systemic inflammation found in cachexia-associated diseases, it would imply that pro-inflammatory bacterial components or metabolites translocate across the gut barrier and would induce a pro-inflammatory systemic response via the GALT. In line with this hypothesis, an increased intestinal permeability has been shown in several cachexia-associated diseases, which could facilitate the crossing of pro-inflammatory molecules through the paracellular pathway (Genton et al., 2015). Furthermore, several randomized controlled trials modulating gut microbiota through pre- pro-, syn-, or antibiotics and demonstrating gut microbiota modifications, also report changes in immune and inflammatory parameters in oncologic surgery (Tanaka et al., 2012), HIV (Pérez-Santiago et al., 2013; Serrano-Villar et al., 2017) and rheumatoid arthritis (Vaghef-Mehrabany et al., 2014). However, no study was performed specifically in cachectic patients.

## Potential Mechanisms Linking Gut Microbiota and Cachexia

**Figure 1:**
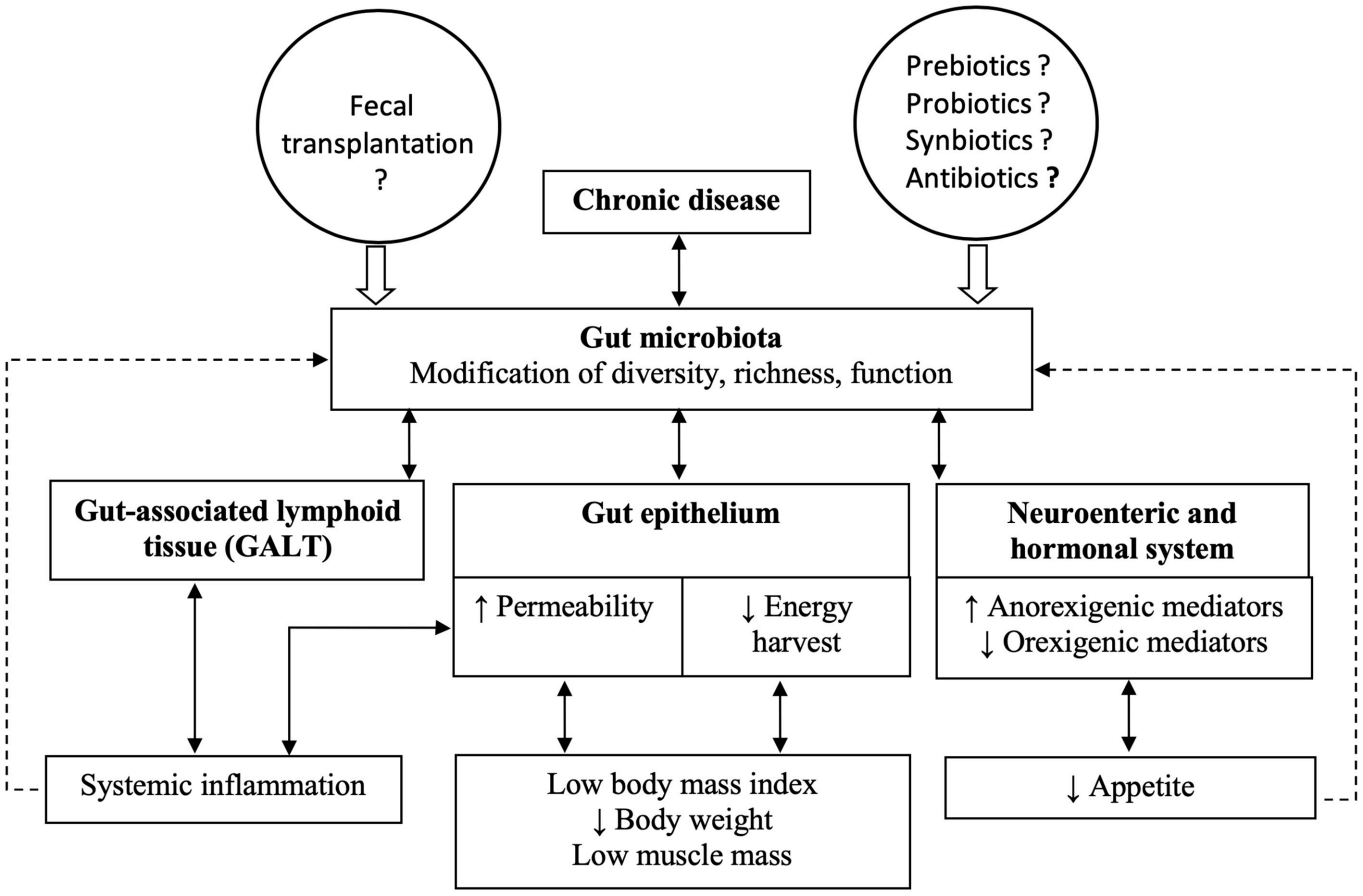
The figure summarizes hypothetical links between chronic diseases and cachexia, based on the opinion of the authors. Chronic diseases lead to changes in gut microbiota composition and function, which in turn affect the components of the gut barrier. The combined modifications of the gut epithelium, the gut-associated lymphoid tissue and the enteric nervous system could result in cachexia.

A simplified scheme summarizes the hypothetical links between chronic diseases and cachexia (Figure 1). As highlighted in the previous paragraphs, one of the hallmarks of disease-related malnutrition is low muscle mass. However, in clinical routine, no method for measuring muscle mass is generally available and it is easier to rely on medical history (body weight loss, anorexia), body weight, and inflammatory plasma markers.

Bindels and Delzenne previously reviewed the potential links between gut microbiota and muscle mass, based on animal studies (Bindels and Delzenne, 2013). Briefly, gut microbiota could lead to low muscle mass through several mechanisms: (1) decreased amino acid bioavailability for the host, because the amino acids are used by the gut microbiota (Lin et al., 2017); (2) stimulation of the Toll-like receptors/NF-kB pathway by pathogen-associated molecular patterns (PAMPs) in muscle cells. Indeed, circulating peptidoglycan of Gram-positive bacteria, lipopolysaccharides, flagellin or bacterial nucleic acids are recognized by the Toll-like receptors in muscle cells, and may activate the NF-kB pathway, which leads to muscle loss (Malavaki et al., 2015); (3) stimulation of pro-inflammatory cytokines secretion resulting from increased gut permeability, described in cachectic diseases, and subsequent translocation of PAMPs from the gut lumen into the GALT, and finally (4) through the production of microbiota-derived metabolites. For instance, bile acids can activate, in the skeletal muscle, the intracellular thyroid hormone leading to an elevation of energy expenditure and the nuclear farnesoid X receptor which hinders fat deposition in the muscle. Furthermore, dietary fibers, converted to short-chain fatty acids by gut microbiota, are involved in body weight control, through increased energy expenditure, appetite control and improved metabolic function of adipose tissue and skeletal muscle in overweight persons (Canfora et al., 2015). By analogy, we could speculate a low amount of short-chain fatty acid production in cachectic patients but this has not been demonstrated.

## Limitations and Perspectives for Human Studies Targeting Gut Microbiota in Cachexia

The afore-mentioned human studies suggest that the fecal material containing the gut microbiota may be involved in energy metabolism and the development of cachexia. However, many issues need to be addressed before using gut microbiota modulation as an evidence-based treatment for cachexia in clinical routine.

Most published studies focused on obesity and not on cachexia. With the increased aging of the population and the increased prevalence of chronic diseases and risk of cachexia, attention should focus also on cachexia, which can be viewed as the opposite metabolic state when based on BMI. As mentioned in the introduction, there is presently no consensus on the definition of cachexia (Table 1). All definitions agree on the components of low BMI and weight loss, although the cut-offs are variable, and some include also low muscle mass and function, anorexia, and systemic inflammation. However, a single definition of cachexia, whatever chronic disease, accepted by all concerned medical specialties, would standardize the patients qualified as cachectic, allow evaluating outcomes with less biases related to the study population and allow to understand if the alterations of gut microbiota and barrier are similar in all diseases associated with cachexia.

Another issue is the lack of a standardized approach to collect, store, process and analyze the feces for bacterial composition and function, which leads to difficult comparisons of results. Standardization of the methodology is essential for future personalized nutrition based on the analysis of the gut microbiota composition and function. Furthermore, most human studies use fecal bacteria as a surrogate marker of gut bacteria. This procedure opens the question whether fecal bacteria reflect the bacterial composition in the lumen of the small bowel, where nutrient digestion and absorption occurs. Also, does the luminal bacterial content which is eliminated in the feces differ from bacteria trapped in the mucus layer of the gut? An insight in the role of bacteria according to its location would require the development of new methods allowing an easy and safe access to the whole small bowel, while presently endoscopy can provide samples only of the proximal small bowel and the colon. Reassuringly, as FMT studies were sufficient to induce weight gain in the recipient, we can hypothesize that in the future we will be able to identify fecal components impacting on the host's health without requiring invasive methods.

Beside the location of the bacteria, and whatever the BMI, it is unclear whether the composition of the gut microbiota, its function, its interactions with other microorganisms, or microbial or host metabolites found in the fecal material are associated with gut barrier alterations and subsequent alterations in energy metabolism. It is likely that other gut components than bacteria, as viruses, protozoans and fungi also play an important role in gut microbial homeostasis and the resulting metabolic health of the host, but unraveling these interactions in cachexia has not yet started. Thus, combined approaches using metagenomics, metabolomics, measurement, or surrogate markers of the gut barrier permeability may provide a better understanding of these associations and subsequently open hypotheses for interventional studies.

Finally, this short review on cachexia components and gut microbiota shows that most knowledge is based on associations and not on causality. Very few longitudinal human studies, whether observational or interventional, have been published. The interpretation of these associations is thus difficult and evidence-based treatments not possible.

## Conclusion

Gut microbiota is an interesting target to potentially treat and prevent cachexia. However, there is presently insufficient evidence that gut microbiota modulation could improve its components and thus clinical outcome in humans.

## Author Contributions

LG, JM, and YC wrote the manuscript. LB, VL, and JS revised the manuscript.

### Conflict of Interest Statement

The authors declare that the research was conducted in the absence of any commercial or financial relationships that could be construed as a potential conflict of interest.
